# Geno- and Phenotypic Characteristics of a *Klebsiella pneumoniae* ST20 Isolate with Unusual Colony Morphology

**DOI:** 10.3390/microorganisms10102063

**Published:** 2022-10-19

**Authors:** Katharina Sydow, Elias Eger, Michael Schwabe, Stefan E. Heiden, Jürgen A. Bohnert, Sören Franzenburg, Christoph Jurischka, Peter Schierack, Katharina Schaufler

**Affiliations:** 1 Pharmaceutical Microbiology, Institute of Pharmacy, University of Greifswald, 17489 Greifswald, Germany; 2 Institute of Infection Medicine, Christian-Albrecht University Kiel and University Medical Center Schleswig-Holstein, 24105 Kiel, Germany; 3 Friedrich Loeffler-Institute of Medical Microbiology, University Medicine Greifswald, 17475 Greifswald, Germany; 4 Institute of Clinical Molecular Biology, Christian-Albrecht University Kiel and University Medical Center Schleswig-Holstein, 24105 Kiel, Germany; 5 Faculty of Environment and Natural Sciences, Brandenburg University of Technology Cottbus-Senftenberg, 01968 Senftenberg, Germany

**Keywords:** *Enterobacterales*, *K. pneumoniae*, virulence, next-generation sequencing, biofilm, exopolysaccharides

## Abstract

*Klebsiella pneumoniae* is a common member of the intestinal flora of vertebrates. In addition to opportunistic representatives, hypervirulent (hvKp) and antibiotic-resistant *K. pneumoniae* (ABR-Kp) occur. While ABR-Kp isolates often cause difficult-to-treat diseases due to limited therapeutic options, hvKp is a pathotype that can infect healthy individuals often leading to recurrent infection. Here, we investigated the clinical *K. pneumoniae* isolate PBIO3459 obtained from a blood sample, which showed an unusual colony morphology. By combining whole-genome and RNA sequencing with multiple in vitro and in vivo virulence-associated assays, we aimed to define the respective *Klebsiella* subtype and explore the unusual phenotypic appearance. We demonstrate that PBIO3459 belongs to sequence type (ST)20 and carries no acquired resistance genes, consistent with phenotypic susceptibility tests. In addition, the isolate showed low-level virulence, both at genetic and phenotypic levels. We thus suggest that PBIO3459 is an opportunistic (commensal) *K. pneumoniae* isolate. Genomic comparison of PBIO3459 with closely related ABR-Kp ST20 isolates revealed that they differed only in resistance genes. Finally, the unusual colony morphology was mainly associated with carbohydrate and amino acid transport and metabolism. In conclusion, our study reveals the characteristics of a *Klebsiella* sepsis isolate and suggests that opportunistic representatives likely acquire and accumulate antibiotic resistances that subsequently enable their emergence as ABR-Kp pathogens.

## 1. Introduction

*Klebsiella pneumoniae* belongs to the *Enterobacterales* and is divided into opportunistic (commensal), hypervirulent (hvKp) and generally antibiotic-resistant (ABR-Kp) subtypes [1]. While the latter mostly affects immunocompromised patients in healthcare settings and causes pneumonia, bacteremia, and urinary tract infections, hvKp can infect healthy individuals leading to severe diseases including liver abscesses and meningitis [1]. Specific virulence characteristics allow hvKp to cause “metastatic” infection in multiple body sites [2]. HvKp are usually classified by hypermucoviscosity, historically characterized by a positive string test, and high siderophore production [3]. However, typing based on specific genetic biomarkers has been shown to be more accurate [4]. Russo et al. proposed *peg-344* (metabolite transporter), *iroB* (salmochelin), *iucA* (aerobactin), and the plasmid-based genes *_p_rmpA* and *_p_rmpA2* (mucoid phenotype regulators) as particular biomarkers [4]. ABR-Kp isolates usually act as opportunistic pathogens but are increasingly difficult to treat due to the exhibition of multiple antibiotic resistance (ABR) features [5]. In addition, in recent years, multiple studies have reported on the emergence of convergent strains combining both hypervirulence and ABR [2,6].

As *K. pneumoniae* are ubiquitous in soil and water, the environment likely represents a reservoir for colonization or infection of humans and animals with these potentially harmful opportunists [7,8,9,10]. Interestingly, several studies have shown that environmental *K. pneumoniae* isolates share high similarities with clinical representatives [11,12,13,14], although capsule types might differ [15]. In addition, *K. pneumoniae* from the environment are usually antibiotic-susceptible, reflecting the broad use of antibiotics in the clinical setting [11]. Once acquired by humans, *K. pneumoniae* may colonize the gastrointestinal tract especially in hospitalized patients, from where it can disseminate into the bloodstream upon epithelial cell wall damage [16]. Following *E. coli*, *K. pneumoniae* is the second leading reason for bloodstream infections [17,18], often in patients suffering from cancer or diabetes mellitus [19].

Here, we investigated a clinical *K. pneumoniae* isolate (PBIO3459) from a blood culture that showed an unusual colony morphology. As it is known that particular phenotypes, e.g., small colony variants [20], reflect bacterial fitness and/or virulence and thus affect potential treatment [21,22], we decided to further investigate our isolate with the altered morphology. To classify PBIO3459, it was phenotypically compared to an archetypal hvKp and a multidrug-resistant (MDR) *K. pneumoniae* isolate with some hypervirulence features (convergent type). Additionally, the isolate was investigated at genomic, transcriptomic and phylogenetic levels.

## 2. Materials and Methods

### 2.1. Sample Isolation and Bacterial Isolates

The sample of interest (PBIO3459) was isolated from a blood culture during routine diagnostic procedures at the University Medicine Greifswald (Germany). The bacterial species *K. pneumoniae* was confirmed by MALDI-TOF MS (VITEK MS, bioMérieux, Marcy l’Etoile, France) and phenotypic antibiotic susceptibility testing (AST) was performed using the VITEK 2 system (bioMérieux, Marcy l’Etoile, France).

Other isolates were used as reference and control for the performed assays: a multidrug-resistant *K. pneumoniae* ST307 isolate from a clonal outbreak in the same university hospital (PBIO1953, [6]), a hypervirulent *K. pneumoniae* ST86 isolate (hvKP1, [23,24]), and an *Escherichia coli* K12 (ST10) (W3110, [25]), as well as an *E. coli* ST131 (IMT18399, [26]). All isolates were stored at −80 °C in lysogeny broth (LB; Carl Roth, Karlsruhe, Germany) containing 20% (*v*/*v*) glycerol (anhydrous; Merck, Darmstadt, Germany).

For initial characterization, PBIO3459 was plated on chromogenic agar (CHROMagar, Paris, France), MacConkey agar (Carl Roth, Karlsruhe, Germany) and on Simmons citrate agar (Carl Roth, Karlsruhe, Germany) supplemented with 5 g/L bile salts (Sigma-Aldrich, St. Louis, MO, USA), 10 g/L *myo*-inositol (Carl Roth, Karlsruhe, Germany) and 10 g/L tryptophan (Sigma-Aldrich, St. Louis, MO, USA). The supplemented Simmons agar showed reliable results for detecting *Klebsiella* species [27].

### 2.2. Whole-Genome Sequencing

A randomly selected single colony from LB agar (LB and 1.5% agar [Carl Roth, Karlsruhe, Germany]) was cultured overnight in 5 mL of LB under shaking conditions (130 rpm) at 37 °C. Total DNA was extracted using the MasterPure DNA Purification Kit for Blood, v.2 (Lucigen, Middleton, WI, USA), according to the manufacturer’s instructions. The isolated DNA was quantified fluorometrically using the Qubit 4 fluorometer and the corresponding dsDNA HS Assay Kit (Thermo Fisher Scientific, Waltham, MA, USA). DNA was sent to the Microbial Genome Sequencing Center (MiGS), now SeqCenter (Pittsburgh, PA, USA), and after library preparation using the Illumina DNA Prep Kit and IDT 10 bp UDI indices (Illumina, San Diego, CA, USA), sequenced on an Illumina NextSeq 2000, producing 2 × 151 bp reads. In addition, DNA was sequenced on the Oxford Nanopore platform to obtain long reads.

### 2.3. Sequence Assembly and Genomic Analyses

Short-read data were trimmed (adapter-trimming, contaminant-filtering, quality-trimming, polymer-trimming) using BBDuk from BBTools v.38.90 (https://sourceforge.net/projects/bbmap/, accessed on 3 March 2022). Read QC of raw and trimmed reads was assessed using FastQC v.0.11.9 (https://www.bioinformatics.babraham.ac.uk/projects/fastqc/, accessed on 3 March 2022). A short-read-only assembly of the trimmed reads was conducted by using the assembly pipeline shovill v.1.1.0 (https://github.com/tseemann/shovill, accessed on 3 March 2022) in combination with SPAdes v.3.15.2 [28] and polished by first mapping the trimmed reads to the assembly using BWA v.0.7.17 [29]. After SAMtools v.1.12-processing (SAM-to-BAM conversion, sorting, duplicate-marking) [30], the draft contigs were corrected using Pilon v.1.23 [31]. Hybrid assembly with short- and long-read data was conducted using Unicycler v.0.4.9 [32] in combination with SPAdes v.3.13.0 [28]. Assemblies were checked for peculiarities in the assembly metrics and additionally assessed using CheckM v.1.1.3 [33] to estimate genome completeness and contamination. Automatic annotation was performed using Prokka v.1.14.6 [34]. In silico multi-locus sequence typing (MLST) and antibiotic resistance and virulence detection were carried out using mlst v.2.19.0 (https://github.com/tseemann/mlst, accessed on 4 March 2022; using the PubMLST database [35]), ABRicate v.1.0.0 (https://github.com/tseemann/abricate, accessed on 4 March 2022; using the ResFinder [36], PlasmidFinder [37], and VFDB [38] databases), and Kleborate v.2.2.0 [39] with Kaptive v.2.0.0 [40].

According to Kleborate, a resistance score of 0 describes the absence of ESBL and carbapenemase genes (a potentially colistin-resistant genotype is not considered in this case). A resistance score of 1 corresponds to the presence of ESBL genes and the absence of carbapenemase genes, whereas a score of 2 indicates the presence of carbapenemase genes without colistin resistance genes (ESBL genes or OmpK mutations are not considered). Accordingly, a resistance score of 3 is characterized by the presence of carbapenemase genes in combination with a colistin-resistant genotype. A virulence score of 0 describes the absence of the siderophores yersiniabactin (*ybt*), colibactin (*clb*), and aerobactin (*iuc*), whereas a score of 1 indicates the presence of only yersiniabactin. A virulence value of 2 corresponds to the presence of yersiniabactin and colibactin (or colibactin only) and 3 describes the presence of aerobactin (without yersiniabactin or colibactin).

To identify genomes of ABR-Kp closely related to PBIO3459, all *K. pneumoniae* GenBank assemblies were downloaded from the National Center for Biotechnology Information (NCBI) Assembly site (accessed on 25 August 2022) using “NCBI:txid573” as the search term. Sequence type (ST) was determined using mlst v.2.19.0. All ST20 genomes (*n* = 293) were analyzed with Kleborate v.2.2.0 to identify genomes with a resistance score ≥ 1. In addition, Mash v.2.3 [41,42] was used to create a sketch archive (sketch size 1,000,000) of PBIO3459 and all ST20 genomes. The pairwise distance from PBIO3459 to all sequences in the sketch archive was then calculated. The Kleborate and Mash results were combined, and the two genomes most closely related to PBIO3459 with a resistance score ≥ 1 (GCA_021917825.1 and GCA_022235845.1) were selected for synteny analysis. Contigs of the two isolates were ordered according to the alignment position to the genome of PBIO3459. Reordered genomes of GCA_021917825.1 and GCA_022235845.1 were annotated using Prokka v.1.14.6. The synteny plot was generated using the gbk files as input for pyGenomeViz v.0.2.1 (mode: pgv-mummer; identity threshold of 50%; https://github.com/moshi4/pyGenomeViz, accessed on 25 August 2022). Genome coverage was calculated based on the align_coords.tsv file. PIRATE v.1.0.4 [43] was used to build a pangenome of the three genomes and to identify the presence and absence of genes.

The downloaded ST20 genomes from NCBI were used to generate a core single-nucleotide polymorphism (SNP)-based phylogeny. Genomes were used as input for snippy v.4.6.0 (https://github.com/tseemann/snippy, accessed on 4 October 2022) with PBIO3459 as reference to create a whole-genome alignment. The alignments were filtered using Gubbins v.3.2.1 [44] to remove recombinations and snp-sites v.2.5.1 [45] was used to extract core SNPs. A maximum likelihood (ML) tree was inferred with RAxML-NG v.1.1.0 [46] using GTR + R. Here, 30 genomes were excluded because they were identical to another sequence. Clustering of the tree was performed using fastbaps [47], with the final multiple sequence alignment and the best-scoring ML tree (mid-point rooted with FigTree [https://github.com/rambaut/figtree, accessed on 4 October 2022]) as input. The clustered tree was midpoint-rooted in iTOL v.6.5.8 [48] and visualized with metadata that was retrieved from the NCBI Assembly and BioSample websites and Kleborate resistance and virulence scores.

### 2.4. RNA Isolation and Sequencing

Randomly selected colonies were picked from chromogenic, LB and brain heart infusion (BHI; Merck, Darmstadt, Germany) agar plates, respectively, and placed into 1 mL of phosphate buffered saline (PBS) each until the optical density measured at *λ* = 600 nm (OD_600_) reached 0.2. Total RNA was extracted using the RNeasy Micro Kit (Qiagen, Hilden, Germany) according to the manufacturer’s instructions. The isolated RNA was checked for purity and quantified using a Qubit 4 fluorometer. RNA was sent on dry ice to the Competence Centre for Genomic Analysis (CCGA, Kiel, Germany) and prepared using the Illumina stranded total RNA kit with Ribozero Plus (Illumina, San Diego, CA, USA). Resulting library preparations were paired-end sequenced (2 × 100 bp reads) using Illumina NovaSeq 6000 (Illumina, San Diego, CA, USA).

### 2.5. Transcriptomic Analysis

Adapter and quality trimming of the raw sequencing reads was performed using Trim Galore v.0.6.7 (https://github.com/FelixKrueger/TrimGalore, accessed on 2 May 2022). The assembly of PBIO3459 was used as reference for mapping the trimmed reads using Bowtie 2 v.2.4.4 [49]. Then, the number of genes was calculated using featureCounts v.2.0.1 [50] based on the annotation of PBIO3459. Differential gene expression was calculated using DESeq2 v.1.36.0 [51] in R v.4.2.0 (https://www.R-project.org/, accessed on 2 May 2022). The analysis was performed in default mode with one exception; genes with rowSums of <10 in the count table were removed prior to the analysis. Genes were considered differentially expressed if the absolute log_2_ fold change was greater than or equal to 2 and the adjusted *p* value was less than or equal to 0.05.

### 2.6. Growth Kinetics

Growth kinetics were measured using a microplate reader (CLARIOstar Plus, BMG LABTECH, Ortenberg, Germany) and performed in LB at 37 °C and 40 °C. Single colonies were picked from LB agar plates and incubated in 5 mL of LB on a rotary shaker at 130 rpm and 37 °C overnight. The bacterial suspension was set to 0.5 McFarland standard turbidity in the respective medium. Kinetics were measured on 96-well plates (Nunc™, Thermo Fisher Scientific, Waltham, WA, USA) and therefore 200 µL of the adjusted bacterial suspension was added to each well. For the kinetics, OD_600_ was measured every 30 min for 24 h, with double-orbital shaking at 200 rpm between the measurements. Each kinetic was performed with three biological and three technical replicates.

### 2.7. Mucoid Phenotype

Hypermucoviscosity was tested using the string test. Therefore, a sterile inoculation loop was placed on a single colony on an agar plate and picked up. If a string of 5 mm or longer formed, the test was defined as positive [52].

The hypermucoviscosity was also tested with a sedimentation assay as described previously [53]. A bacterial suspension with a turbidity according to the 0.5 McFarland standard was prepared in 0.9% (*w*/*v*) NaCl solution. From this suspension, 50 µL were added to 5 mL of LB and incubated on a rotary shaker at 130 rpm and 37 °C for 24 h. After incubation, 1.5 mL of the cultures were given in 2 mL reaction tubes (Carl Roth, Karlsruhe, Germany) and centrifuged at 1000× *g* for 5 min at room temperature (RT). Then, 200 µL of the supernatant and 200 µL of the initial culture were transferred separately into a 96-well plate and the OD_600_ was measured. The assay was only performed with *K. pneumoniae* isolates.

The ability to produce exopolysaccharides was tested using BHI agar plates supplemented with 5% (*w*/*v*) sucrose (Carl Roth, Karlsruhe, Germany) and 0.08% (*w*/*v*) congo red (Carl Roth, Karlsruhe, Germany) as previously described [54]. A single colony was taken from an agar plate, streaked onto the BHI agar plates, and then incubated overnight at 37 °C. The assay was done with the *K. pneumoniae* isolates with addition of PBIO1961 (*K. pneumoniae*) and PBIO2010 (*K. pneumoniae*) as positive and negative control, respectively. Exopolysaccharide producers would show black colonies while non-producers would show orange to white colonies.

### 2.8. Serum Resistance

To determine the survival in human blood, serum resistance was tested in 50% human serum as described elsewhere [53]. Overnight cultures were diluted 1:100 with fresh LB and incubated on a rotary shaker at 130 rpm and 37 °C until the OD_600_ reached 0.5 McFarland standard turbidity. From this suspension, 1 mL was given into a 1.5 mL reaction tube (Carl Roth, Karlsruhe, Germany) and centrifuged at 7500× *g* for 5 min at RT. The supernatant was discarded and the pellet was resuspended in 1 mL of PBS. On a 96-well plate, 100 µL of the bacterial suspension were mixed with 100 µL of human serum (US origin, Sigma-Aldrich, St. Louis, MO, USA). Then, 10 µL were taken from each well and serial dilutions were plated on LB agar plates to quantify the colony forming units (CFUs) per mL in the inoculum. The 96-well plate was incubated at 37 °C for 4 h. A second serial dilution was performed to determine the number of survived CFUs/mL. All LB agar plates were incubated overnight at 37 °C and formed colonies were counted the next day. Serum-resistant hvKP1 (*K. pneumoniae* ST86) was used as positive control and serum-sensitive W3110 (*E. coli* K12 ST10) was used as a negative control.

### 2.9. Siderophore Production

The ability to produce siderophores was tested using a previously described method [53]. Briefly, bacterial cultures were again set to 0.5 McFarland standard turbidity in 0.9% (*w*/*v*) NaCl solution and diluted 1:100 in 5 mL of iron-chelated M9 medium (200 μM 2,2′-dipyridyl [Carl Roth, Karlsruhe, Germany] added M9 minimal salt medium [MP Biomedicals, Irvine, CA, USA]) supplemented with 0.3% (*w*/*v*) casamino acids (c-M9-CA, BD, Franklin Lakes, NJ, USA) in sterile 15 mL tubes (Sarstedt, Nürnbrecht, Germany). After incubation on a rotary shaker at 130 rpm and 37 °C for 24 h, 1 mL of sample was given into 1.5 mL reaction tubes and centrifuged for 20 min at 4900× *g* and RT. A 96-well plate was prepared with 100 µL of Chromazurol S (CAS) shuttle solution (composited according to [55]) and 100 µL of the supernatant were added. Fresh media served as blank and 15 mM aqueous EDTA solution (Carl Roth, Karlsruhe, Germany) as a positive control. After incubation in the dark for 30 min at RT, the OD was measured at *λ* = 630 nm. Secretion of siderophores was calculated as previously described [56] and expressed as a percentage unit of siderophore production. The non-siderophore producing *E. coli* isolate (W3110) was used as a negative control.

### 2.10. Long-Term Colonies, Cellulose Production and Biofilm Formation

To test the production of cellulose and/or curli fimbriae, a long-term colony assay was performed as described previously [57]. Briefly, 20 mL of dye (containing 50 mg congo red and 25 mg Coomassie-Brilliant-Blue G-250 [Carl Roth, Karlsruhe, Germany] dissolved in deionized water and ethanol [99%, *v*/*v*; 1:7]) was filter sterilized into 1 L of LB Lennox (Carl Roth, Karlsruhe, Germany) containing 1.8% (*w*/*v*) span agar (Hellmuth Carroux, Hamburg, Germany). From overnight cultures, 5 µL were dropped onto the agar plates. The plates were hermetically sealed and incubated at 28 °C for 5 days followed by visual evaluation. Purple colonies would mark the production of curli fimbriae, structured areas indicated the production of cellulose and white colonies did not show any of the structures [58].

Cellulose production was also tested using a calcofluor assay in 24-well microtiter plates (Sarstedt, Nürnbrecht, Germany). Calcofluor has a high affinity for cellulose and allows the quantification of cellulose production [59,60]. For this purpose, LB Lennox was prepared with 1.8% (*w*/*w*) span agar. An aqueous 0.2% (*w*/*v*) calcofluor solution (Sigma-Aldrich, St. Louis, MO, USA) was sterile filtered and 2 mL were added to 100 mL of the span agar solution. Of this solution, 1 mL was transferred into each well of the 24-well plate and allowed to harden. Colonies from agar plates were taken and added to sterile PBS until OD_600_ = 0.5 was reached and 5 µL of the suspension were dropped onto the center of each well. After 5 days of incubation at 28 °C, the fluorescence intensity was measured using the microplate reader (excitation 400–415 nm, emission 480–520 nm).

Biofilm formation was tested with a crystal-violet (CV) assay [26]. From overnight cultures in LB, a 1:100 dilution in M9 medium supplemented with 1 mM magnesium sulfate (Carl Roth, Karlsruhe, Germany) and 0.4% glucose (Carl Roth, Karlsruhe, Germany) was prepared and incubated on a rotary shaker at 130 rpm and 37 °C until 0.5 McFarland standard turbidity was reached. The OD was then set to 0.01 in the supplemented M9 medium and 200 µL of this suspension were placed into a 96-well plate, which was incubated at 28 °C for 24 h. Additionally, triplicates of 200 µL of sterile medium were used as controls and blanks. The OD_600_ was then measured using the microplate reader. The 96-well plate was washed thrice with deionized water to remove planktonic cells and air-dried for 10 min. Then, the cells were fixed in 250 µL of methanol (Merck, Darmstadt, Germany) for 15 min. After air-drying, cells were stained with 250 µL of a 0.1% (*w*/*v*) aqueous CV solution (Sigma-Aldrich, St. Louis, MO, USA) for 30 min, before the plate was washed three times with deionized water and dried for 10 min. Subsequently, the bound CV was dissolved in 300 µL of a mixture of 80 parts ethanol (99.8% (*v*/*v*); Carl Roth, Karlsruhe, Germany) and 20 parts acetone (Merck, Darmstadt, Germany) at RT with horizontal shaking at 200 rpm for 30 min. Finally, 125 µL of this solution were transferred to a new 96-well plate and the OD was measured at *λ* = 570 nm using the microplate reader. The strength of biofilm formation was expressed as specific biofilm formation (SBF). The SBF was calculated according to the following formula [61]: SBF = (B − NC)/G, where B is the OD_570_ of the stained bacteria, NC is the OD_570_ of the stained control wells to eliminate the fraction of CV adhering to the polystyrene surface due to abiotic factors, and G is the OD_600_ representing the density of cells grown in the media.

W3110 and the biofilm-producing IMT18399 were used as controls. W3110 was used as a negative control for cellulose production, while both isolates were used as positive controls for the other assays.

### 2.11. Infection of Galleria mellonella Larvae

The mortality of the *K. pneumoniae* isolates (PBIO1953, hvKP1, PBIO3459) was tested using larvae of the wax moth *Galleria mellonella* as model organisms, as described previously [62]. Several single colonies were suspended in PBS until an OD_600_ of 1.0 was reached (approx. 2 × 10^9^ CFU/mL). The bacterial suspensions were then centrifuged at 12,000× *g* and RT for 5 min and washed twice with PBS. The suspension was diluted to 2 × 10^7^ CFU/mL. Larvae (proinsects, Minden, Germany) were randomly divided into groups of 10 individuals each and 10 µL of the adjusted bacterial suspensions were injected into the left proleg. In addition, 10 µL of PBS was injected into a group of larvae to ensure that death was not due to trauma from the injection. Each group was placed in 90 mm glass Petri dishes, kept at 37 °C in the dark, and death was recorded every 24 h. Individuals were considered dead when they no longer responded to physical stimuli and showed pigmentation. The assay was repeated three times, results for each isolate were pooled and Kaplan–Meier plots were generated to show mortality rates [63].

### 2.12. Data Visualization and Statistical Analyses

Statistical analyses were performed using GraphPad Prism v.7.05 for Windows (GraphPad Software, San Diego, CA, USA). All phenotypic experiments were performed with three biological replicates. Unless otherwise indicated, data were expressed as mean and standard deviation. Statistical significance was assessed by analysis of variation (ANOVA) with Dunnett’s multiple comparison *post hoc* test. *p* values of less than 0.05 were used to indicate significant statistical differences among the results.

## 3. Results

### 3.1. PBIO3459 Lacks Typical Antibiotic Resistance and Hypervirulence-Associated Genes

**Figure 1:**
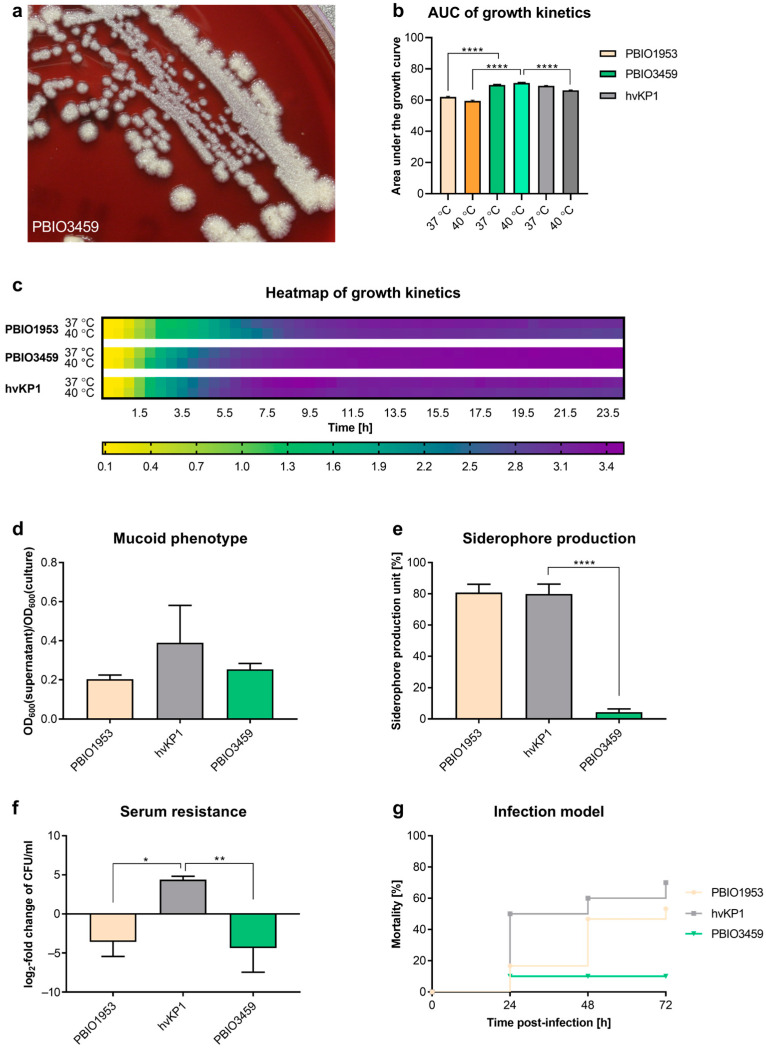
Phenotypic virulence-associated features of PBIO3459. (**a**) PBIO3459 on blood agar with sponge-like colonies. (**b**,**c**) Heatmap of growth kinetics in LB at different temperatures and corresponding area under the growth curve (AUC) (*n* = 3). The results are given as mean values with standard deviation. (**d**) Determination of the mucoid phenotype using a sedimentation assay (*n* = 3). The results are given as mean ratios of OD_600_ of supernatant after centrifugation at 1000× *g* for 5 min and total OD_600_ and standard deviation. (**e**) Siderophore production of the respective isolates, shown as siderophore production unit (spu) and standard deviation (*n* = 3). (**f**) Survival in 50% human serum after 4 h of incubation. Results are given as mean values and standard deviation of log_2_ fold change of CFU/mL (*n* = 3). (**g**) Kaplan–Meier plot of mortality rates in the Galleria mellonella larvae infection model (*n* = 3). Results are given as mean mortality after injection of 2 × 10^5^ CFU. Statistical significance was tested with one-way ANOVA and (for (**g**)) two-way ANOVA (with Dunnett’s multiple comparison *post hoc* test); * *p* < 0.05, ** *p* < 0.01, **** *p* < 0.0001.

PBIO3459 was isolated from a blood sample of a patient with diabetes mellitus and gastroenteritis and exhibited colony morphology unusual for *Klebsiella* (Figure 1a). On blood, MacConkey, and chromogenic agar, colonies demonstrated a rough, dry, and elevated surface (Figure 1a), whereas on LB and supplemented Simmons agar, colonies with a smooth and even surface were observed. Species identification using MALDI-TOF MS revealed PBIO3459 as *K. pneumoniae*. Whole-genome sequence (WGS) analysis showed that PBIO3459 belonged to ST20 and capsule biosynthesis (KL) and lipopolysaccharide antigen (O) loci were KL28 and O1/O2v2, respectively. Interestingly, the insertion sequence (IS) *ISKPn74*, previously described in a hvKp isolate [53], was found within the K locus (identity: 1050/1056 bp, 99%). In addition, we identified *IS903B* (identity: 1029/1057 bp, 97%) within the O locus and in the region between both loci. PBIO3459 carried a plasmid with incompatibly group (Inc)FIA and Col-plasmids but no acquired antibiotic resistance genes, which was confirmed by AST as only intrinsic ampicillin and amoxicillin phenotypic resistances were detected.

Using VFDB to assign virulence factors, overall 109 different genes were identified. The most abundant genes were associated with metabolic factors (*n* = 29), immune modulation (*n* = 22), adherence (*n* = 19), and effector delivery systems (*n* = 18). The metabolic factors were mainly related to iron uptake and transport such as the two siderophore systems yersiniabactin (*fyuA*, *irp1*, *irp2*, *ybtAEPQSTUX*), and enterobactin (*entABCDF*, *fepABCDG*, *fes*). In addition, some genes encoding for salmochelin (*iroEN*) and aerobactin (*iutA*) siderophores were present, but we did not detect the complete systems. Adherence genes were mainly related to fimbriae and pili and all genes associated with effector delivery systems encoded for the type VI (14/18) and type II secretion systems (4/18).

### 3.2. PBIO3459 Demonstrates General Low Virulence but Increased Biofilm Formation

To gain further insights regarding the assignation of PBIO3459 to a particular subtype, it was tested in phenotypic fitness-, virulence-, and resilience-associated assays (Figure 1) and compared to different reference strains, i.e., an archetypical hypervirulent (hvKP1) and an extensively drug-resistant *K. pneumoniae* with enhanced virulence (convergent strain PBIO1953).

Regarding basic growth at two different temperatures, aiming at testing healthy (37 °C) as well as diseased (fever) conditions (40 °C), the three isolates showed similar kinetics in LB medium (Figure 1b,c). However, comparing the area under the curve (AUC) at 37 °C, the AUC of PBIO1953 (62.05, *p* < 0.0001) was significantly lower than that of PBIO3459 (69.73), while it was not significant for hvKP1 (69.1) compared to PBIO3459 (*p* = 0.37). Comparison of the AUCs at 40 °C showed that the AUC of PBIO3459 (70.96) was significantly higher than those of hvKP1 (66.31, *p* < 0.0001) and PBIO1953 (59.48, *p* < 0.0001) (Figure 1b).

Next, we tested whether PBIO3459 demonstrated any typical hypervirulence-associated features. In the hypermucoviscosity string test, only hvKP1 colonies formed a string longer than 5 mm and were thus defined positive. For PBIO1953 and PBIO3459, the string test was negative. Second, we performed a sedimentation experiment. Mucus production affects the sedimentation behavior of cells as mucus is more viscous than the surrounding medium. During centrifugation, mucus-producing cells sediment more slowly due to the viscous properties. The ratio between the supernatant and the OD_600_ of the complete culture can be calculated and compared with the different isolates to assess hypermucoviscosity. The highest mean ratio was observed for hvKP1 (0.39). Ratios of PBIO3459 (0.25; *p* = 0.30) and PBIO1953 (0.20; *p* = 0.15) were lower but not statistically significant (Figure 1d). Similar results were obtained for siderophore production, where high values were measured for PBIO1953 and hvKP1, while PBIO3459 produced a significantly lower amount (*p* < 0.0001) (Figure 1e). This matches the WGS data, where only yersiniabactin and enterobactin siderophore systems were found, while aerobactin, the dominant siderophore in hvKp [64], was absent. When we challenged the isolates with 50% human serum, both PBIO3459 (*p* = 0.0085) and PBIO1953 (*p* = 0.0143) showed significantly lower CFU levels than hvKP1 after 4 hours of incubation (Figure 1f). Finally, to explore the isolates’ ultimate virulence potential, we performed in vivo mortality in *Galleria mellonella* larvae (Figure 1g). As expected, hvKP1 showed the highest mortality rate (50.0% after 24 h; 60.0% after 48 h; 70.0% after 72 h) whereas PBIO3459 killed significantly lower numbers of larvae (10.0% after 24 h, *p* = 0.0004; 10.0% after 48 h, *p* < 0.0001; 10.0% after 72 h, *p* < 0.0001). Except for the time point after 24 h, the mortality rate of PBIO3459 was also significantly lower than that of PBIO1953 (16.7% after 24 h, *p* = 0.69; 46.7% after 48 h, *p* = 0.0010; 53.3% after 72 h, *p* = 0.0002).

**Figure 2:**
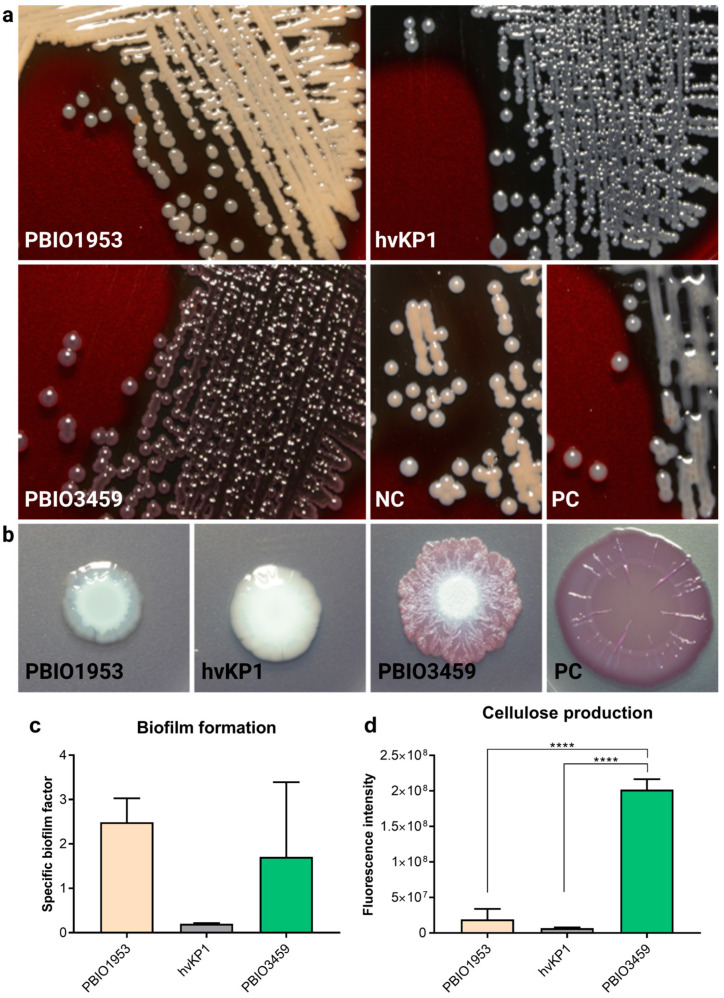
Representative images of phenotypic assays and phenotypic assays related to biofilm forming abilities and pathogenesis. (**a**) Production of exopolysaccharides. Isolates were plated on BHI agar and incubated for 24 h at 37 °C. PBIO1953 was tested negative for the production of exopolysaccharides, hvKP1 was tested positive and the result for PBIO3459 could not be determined clearly. The negative control (NC) was tested negative and the positive control (PC) positive. (**b**) Cellulose and curli fimbriae production was investigated in long-term colonies. PBIO1953 and hvKP1 produced neither structures while the phenotype of PBIO3459 differed from usual (control) long-term colonies. The PC produced both curli fimbriae and cellulose. (**c**) Adherence ability given as specific biofilm factor. Results are given as mean values with standard deviation. (**d**) Cellulose production of the respective isolates. Results are expressed as mean fluorescence intensities with standard deviation (*n* = 3). Statistical significance was tested with one-way ANOVA (with Dunnett’s multiple comparison *post hoc* test); **** *p* < 0.0001.

As the unusual rough appearance of PBIO3459 on certain nutrient media might be associated with modified capsular polysaccharides, the production of exopolysaccharides was investigated using BHI agar supplemented with sucrose and congo red (Figure 2a). Upon exopolysaccharides production, the respective bacterium grows in grayblackish colonies, whereas orange and white colonies indicate no exopolysaccharide production. PBIO1953 was tested negative for exopolysaccharides showing orange colonies; hvKP1 grew in gray-blackish colonies and was therefore tested positive. The result of PBIO3459 could not be determined clearly, as colonies showed a red-grayish color.

In addition to capsular components, many *Klebsiella* isolates form biofilms, which have been previously related to intestinal colonization and infection. Here, we tested the production of important biofilm-associated cellulose and curli fimbriae structures in long-term colonies. PBIO1953 and hvKP1 were white and unstructured and thus classified negative for curli and cellulose production, whereas colonies of PBIO3459 could not be assigned to any particular phenotype as they appeared differently than the positive control (Figure 2b). To further explore the biofilm-associated results, we performed a crystal violet experiment that tested the isolates’ ability to adhere to plastic surfaces, revealing that PBIO1953 and PBIO3459 had similar specific biofilm formation capacities whereas hvKP1 had a lower adhesion affinity. However, note that these differences were not statistically significant (Figure 2c). Cellulose production was tested additionally, using the fluorescent dye calcofluor. In summary, the assay suggests PBIO3459 as an extensive cellulose producer (Figure 2d).

### 3.3. PBIO3459 Is Closely Related to Antibiotic-Resistant Klebsiella

**Figure 3:**
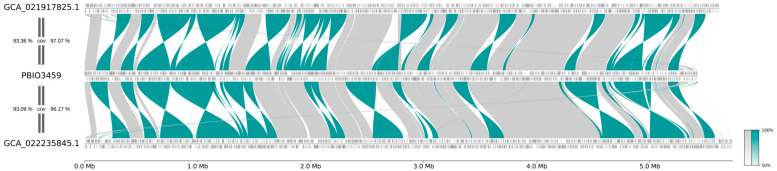
Synteny plot of PBIO3459 compared to most related, publicly available genomes of MDR *K. pneumoniae* ST20 strains. Genomes were selected based on a Kleborate resistance score ≥ 1 and most shared k-mers using a Mash-based approach. The synteny plot shows a large extent of similar (gray) or inverted regions (teal) of the draft assembly PBIO3459 and the selected genomes. cov: genome coverage.

To address the genomic relatedness of antibiotic-resistant ST20 isolates and PBIO3459, we selected two publicly available *K. pneumoniae* ST20 genomes based on the following two criteria: (i) a Kleborate resistance score of ≥1 and (ii) the most shared k-mers using a Mash-approach. Like PBIO3459, the two identified genomes GCA_021917825.1 and GCA_022235845.1 demonstrated the K/O loci KL28 (for the former unknown KL28) and O1/O2v2, respectively, and a virulence score of 1. Kleborate revealed a resistance score of 1 for GCA_021917825.1 with 14 resistance genes conferring resistance to six antibiotic classes (aminoglycosides, quinolones, sulfonamides, trimethoprim, beta-lactams, including third-generation cephalosporins and monobactams). In contrast, GCA_022235845.1 had a resistance score of 2 and exhibited seven resistance genes to three antibiotic classes (quinolones, beta-lactams, including carbapenems). When comparing the two genomes to PBIO3459, we noticed high sequence similarities, reflected by high genomic coverage (Figure 3). GCA_021917825.1 and GCA_022235845.1 sequences both covered over 96% of the complete genome of PBIO3459 whereas, in contrast, PBIO3459 covered approximately 93% of the draft genomes of GCA_021917825.1 and GCA_022235845.1 (Figure 3) suggesting that all three genomes possessed unique genomic areas, e.g., mobile genetic elements. To investigate these slight differences at the gene level, we performed group clustering using PIRATE. Most groups (83.77%; 4739/5657) were found in all three genomes, while we detected only 1.77% (100/5657) exclusively in PBIO3459, and 4.40% (249/5657) and 6.15% (348/5657) were present in GCA_021917825.1 and GCA_022235845.1 only. Most unique genes were hypothetical proteins (HP). Interestingly, in addition to HP, heavy metal resistance (HMR) and heat shock proteins were found in the GCA genomes. Twenty-four percent (24/100) of the unique groups in PBIO3459 were of plasmid origin and another 25% (25/100) were associated with prophages, which are not uncommon in human-associated, clinical strains [65]. In summary, the investigated ST20 genomes only differed in the presence of ABR and HMR genes.

**Figure 4:**
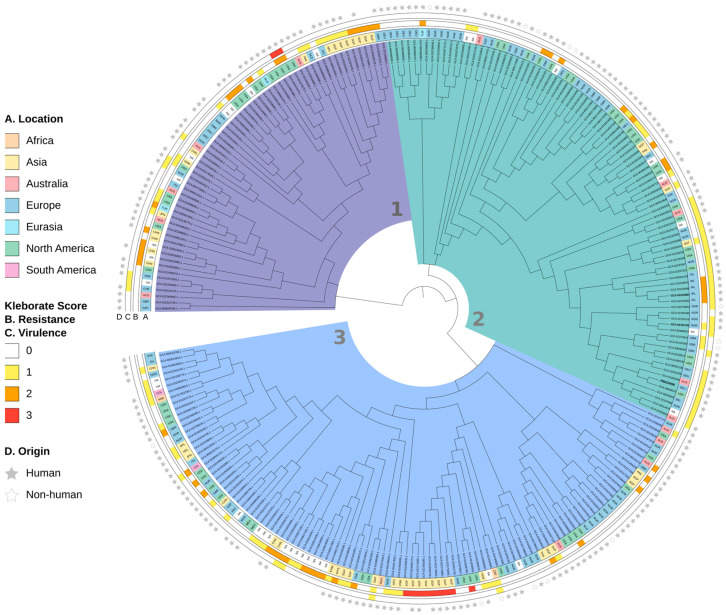
Phylogenetic tree of *K. pneumoniae* ST20 genomes using a ML approach based on SNP alignments. The labels indicate the accession number (except PBIO3459). Annotations indicate (from inner circle to outer circle): isolate location (A., continents), Kleborate resistance and virulence scores (B. + C.), and isolate human or non-human origin (D.). Countries are abbreviated with alpha-3 codes; *NA*: not applicable; absent star: not applicable (i.e., no metadata provided). Genomes used for the synteny plot are highlighted in bold.

Then, to investigate our isolate in a global context, we performed a phylogenetic analysis for which we used all publicly available ST20 genomes and PBIO3459 (Figure 4).

The dataset included 264 international genomes from 35 countries. The majority originated from Europe (37.5%; 98/264), North America (23.11%; 61/264), and Asia (18.56%; 49/264) and was isolated from humans (211/264).

The phylogenetic tree, which is based on 8904 core SNP sites, contained three main clades. While clade 3, as the largest, consisted of 110 genomes, clade 2 contained 92, and clade 1 62 genomes, respectively. Most genomes with a resistance score of ≥1 and low virulence levels belonged to clades 1 and 3. Interestingly, two clade 1-genomes (GCA_021973675.1 and GCA_022470475.1) from North America demonstrated high resistance (score = 2) and virulence (score = 3) levels, suggesting convergent *Klebsiella* types. Within clade 3, nine partially unrelated genomes showed an extensively drug-resistant genotype (resistance score 3, virulence score 0). The intra-clade distance, i.e., the number of SNPs between the two closest as well as the most distant genomes within one clade, was 2–291 (median distance: 187) for clade 1 and 1–303 (median distance: 182) for clade 3.

Clade 2, containing PBIO3459, had an intra-clade distance of 1–190 (median distance: 104). Also note that clade 2 divided into two sub-clusters, both with genomes demonstrating resistance scores between 0 and 2. The first cluster consisted of genomes with a virulence score of 0, whereas the second mainly contained genomes with a virulence score of 1. PBIO3459 belonged to the latter and was closely related to a ST20 genome from North America (GCA_008082375.1) that is also human-derived.

### 3.4. PBIO3459 Reveals Up-Regulation of Extracellular Sugar Degradation-Associated Genes

Finally, to explore potential underlying mechanisms of the two different phenotypes, RNA sequencing was performed. As PBIO3459 showed the same (smooth) phenotype on LB and BHI agar, both conditions were compared with the other phenotype (rough) that occurred on chromogenic agar. Of particular interest were those genes that were down-regulated in the rough phenotype whilst up-regulated in the smooth phenotype and vice versa. Therefore, a double comparison was done. Genes that were differentially expressed on chromogenic agar vs. LB agar were compared with chromogenic agar vs. BHI agar. We identified nine down-regulated and 48 up-regulated genes on chromogenic agar compared with both LB and BHI (Figure S1). According to the database of clusters of orthologous groups [66], the most abundant groups within the down-regulated genes were associated with amino acid transport and metabolism (3/9) and transcription (2/9). Most up-regulated genes were related to carbohydrate transport and metabolism (14/48) or energy production and conversion (7/48). The differentially expressed genes were further investigated using the Kyoto encyclopedia of genes and genomes (KEGG) database [67,68,69]. The down-regulated genes did not show clear clustering regarding particular pathways but some of the genes were related to lysine metabolism (*mdcR*, *lysA*, *cadB*). For the up-regulated genes, the KEGG database detected many genes potentially involved in starch and sucrose metabolism. In particular, genes associated with the degradation of extracellular sugars and glucose production were found to be up-regulated. In this context, *chbA*, *celAB*, *gmuB*, *bglBCF* (degradation of extracellular cellobiose to glucose) and *treBC* (degradation of extracellular trehalose to glucose-6-phosphate) were particularly noteworthy. A similar pattern of up-regulated genes was also seen when only comparing the data of chromogenic agar with LB agar. Many sugar-related genes, e.g., associated with maltoporins, mannose, galactosidases, glucosidases and cellobiose, were up-regulated, whereas amino acid-related genes were down-regulated, e.g., associated with lysine, leucine, cysteine, and methionine. However, it was not possible to identify unique genes associated with either the smooth or the rough phenotype when comparing only two media, as pure media effects could not be excluded.

## 4. Discussion

To investigate the virulence and ABR potential of PBIO3459 and provide a subsequent classification, several phenotypic assays were performed in addition to whole-genome and RNA sequencing. The rough colonies of PBIO3459 on chromogenic media had a sponge-like appearance, similar to rugose colony phenotypes of *Nocardia* spp. [70,71] or *Vibrio cholerae* [72]. To our knowledge, such morphologies have not been previously reported for *K. pneumoniae*. While PBIO3459 showed low virulence in hypermucoviscosity (string and sedimentation tests), siderophore production and serum resistance assays, as well as in vivo mortality, which are all traits typical for hvKp, good production of exopolysaccharides and cellulose as well as biofilm formation revealed characteristics putatively important for intestinal colonization and thus opportunistic pathogens [73]. WGS analysis revealed that only two minor siderophore systems (yersiniabactin and enterobactin) were present, resulting in the observed low siderophore production unusual for hvKp isolates [74,75]. Serum resistance of PBIO3459 was also low, which again seems logical when classifying it as opportunistic pathogen that lacks the appropriate virulence features. Indeed, hvKP1 demonstrated the opposite phenotype. It has been previously reported that the lipopolysaccharide composition of outer membrane components [76] but also the siderophore aerobactin [64] play important roles in bacterial serum resistance. Changes in those lipopolysaccharides [76] or deletion of aerobactin can lead to reduced growth in serum [64], supporting our results. Overall, concluding our phenotypic results and the absence of specific genetic biomarkers, PBIO3459 seemingly does not belong to the hvKp pathotype. Since it also does not meet the criteria for being classified as MDR representative, as supported by an antibiotic resistance score of 0 and phenotypic AST, the opportunistic/commensal character of PBIO3459 is highly likely. We speculate that PBIO3459 disseminated to the bloodstream from the intestinal tract, enabled by the patient’s severe gastroenteritis and subsequent potentially disrupted blood–intestinal barrier.

Interestingly, PBIO3459 belongs to a phylogenetic background (ST20) that can cause fatal infections. Outbreaks with MDR ST20 isolates have been reported in several countries such as South Korea [77], Spain [78,79], Canada [80], New Zealand [81], Greece [82], Brazil [83], and China [84,85,86,87,88,89,90,91], and isolates without ABR have occasionally been reported [77,82,83,91]. In a few cases, *K. pneumoniae* ST20 were isolated from healthy individuals [92] or those without an underlying infection [82]. For example, Lepuschitz et al. investigated the colonization pattern of *K. pneumoniae* in healthy individuals [92]. They found a variety of different STs, including ST20 isolates; one ST20 KL102 O2v2 and one ST20 KL28 O1v2 isolate [92], with the latter demonstrating the same K and O loci as PBIO3459, again supporting its classification. Mavrodi et al. reported an outbreak in neonates caused mainly by *K. pneumoniae* ST20 [82]. They identified ST20 isolates in infected but also colonized neonates [82]. It is well known that ABR can spread through mobile genetic elements and that commensal microorganisms acquire such traits in the intestinal tract [93]. For example, a ST20-K28 *K. pneumoniae* isolate carrying a hybrid plasmid with the potential for horizontal gene transfer has been previously reported [90]. Additionally, here, we revealed high genome sequence identities of exemplary ST20 isolates, only differing in the presence of ABR and HMR genes. The latter follows in the context of their frequently described co-localization on mobile genetic elements [94]. In addition, we revealed PBIO3459′s close relationship (median intra-clade distance: 104) to ST20 genomes from different European locations and worldwide. Of the 14 genomes most closely related to PBIO3459, eleven were of human, and two from wastewater origin (Table S1). In addition, note that the combination of genomic resistance and virulence features present in the two isolates from North America might suggest their convergent character. However, this will have to be further explored in the future. Taken together, our results suggest that ABR-Kp isolates may descend from intestinal commensals by acquiring resistance, possibly then leading to difficult-to-treat infections.

Finally, our RNA sequencing analysis to explore the underlying mechanisms of PBIO3459′s two different phenotypes revealed several pitfalls. Since the exact composition of chromogenic agar is unknown, it was not possible to confidently relate the results to meaningful biological processes and exclude media effects. LB agar only contains amino acids as nutrients and no sugars [95]. Therefore, when LB is compared to another medium which contains sugars as nutrients, the up-regulation of sugar-associated genes is a logical consequence, while noticing a down-regulation of amino acid-associated genes. Some sugar-associated genes, e.g., for mannose, fucose, rhamnose or galactofuranose, could be related to capsular polysaccharide synthesis [96], but these genes could either be phenotype- or simply medium-related. However, as PBIO3459 showed the same phenotype (smooth) on LB and BHI agar, while rough colonies appeared on chromogenic medium, we investigated genes that were up- or down-regulated on both, LB and BHI agar, in comparison to chromogenic agar to receive actually meaningful data. Therefore, the up-regulated genes associated with the degradation of extracellular sugars and glucose production might be responsible for the different phenotypes indeed. As extracellular polysaccharides can be used as nutrient reservoirs [97], depletion or lack of available nutrients might lead to the degradation of PBIO3459′s extracellular sugars. Cellulose, as an architectural polysaccharide, might also contribute to the phenotype [97]. Cellulose is, i.a., involved in the expression of the multicellular rdar (rough, dry, and red) morphotype of *Salmonella typhimurium* and some *E. coli* isolates [97,98]; however, these phenotypes do not exactly match our isolate. Another architectural polysaccharide potentially leading to rough colony morphologies is colanic acid, which is frequently formed at low temperatures [97,99]. Interestingly, deletion mutants of *E. coli* have shown the importance of colanic acid in the formation of complex three-dimensional structures [100]. As we have performed all biofilm-related assays at 28 °C, colanic acid might also be a contributing factor. However, this issue needs to be addressed prospectively.

## 5. Conclusions

Here, we investigated a *K. pneumoniae* isolate with unusual colony morphology from a blood sample and revealed its low-level antibiotic resistance and virulence features and thus opportunistic character. In addition, we discussed potential underlying mechanisms contributing to the phenotype and the probability that ABR-Kp originate from opportunistic representatives through the acquisition of antibiotic resistance traits.

## Data Availability

The data for this study have been deposited in the European Nucleotide Archive (ENA) at EMBL-EBI under accession number PRJEB55904 (https://www.ebi.ac.uk/ena/browser/view/PRJEB55904, uploaded on 13 September 2022).

## Supplementary Materials

The following supporting information can be downloaded at: https://www.mdpi.com/article/10.3390/microorganisms10102063/s1, Figure S1: Summary of the differential gene expression; Table S1: Metadata for *K. pneumoniae* ST20 genomes used for phylogenetic analysis.
