# Supplementary material for: Geno- and Phenotypic Characteristics of a Klebsiella pneumoniae ST20 Isolate with Unusual Colony Morphology
Source: Microorganisms. 2022 Oct 19;10(10):2063. doi: 10.3390/microorganisms10102063 (PMC9606995; doi:10.3390/microorganisms10102063)
Supplement: Supplementary file 1 [file microorganisms-10-02063-s001.zip › Figure S1.pdf]

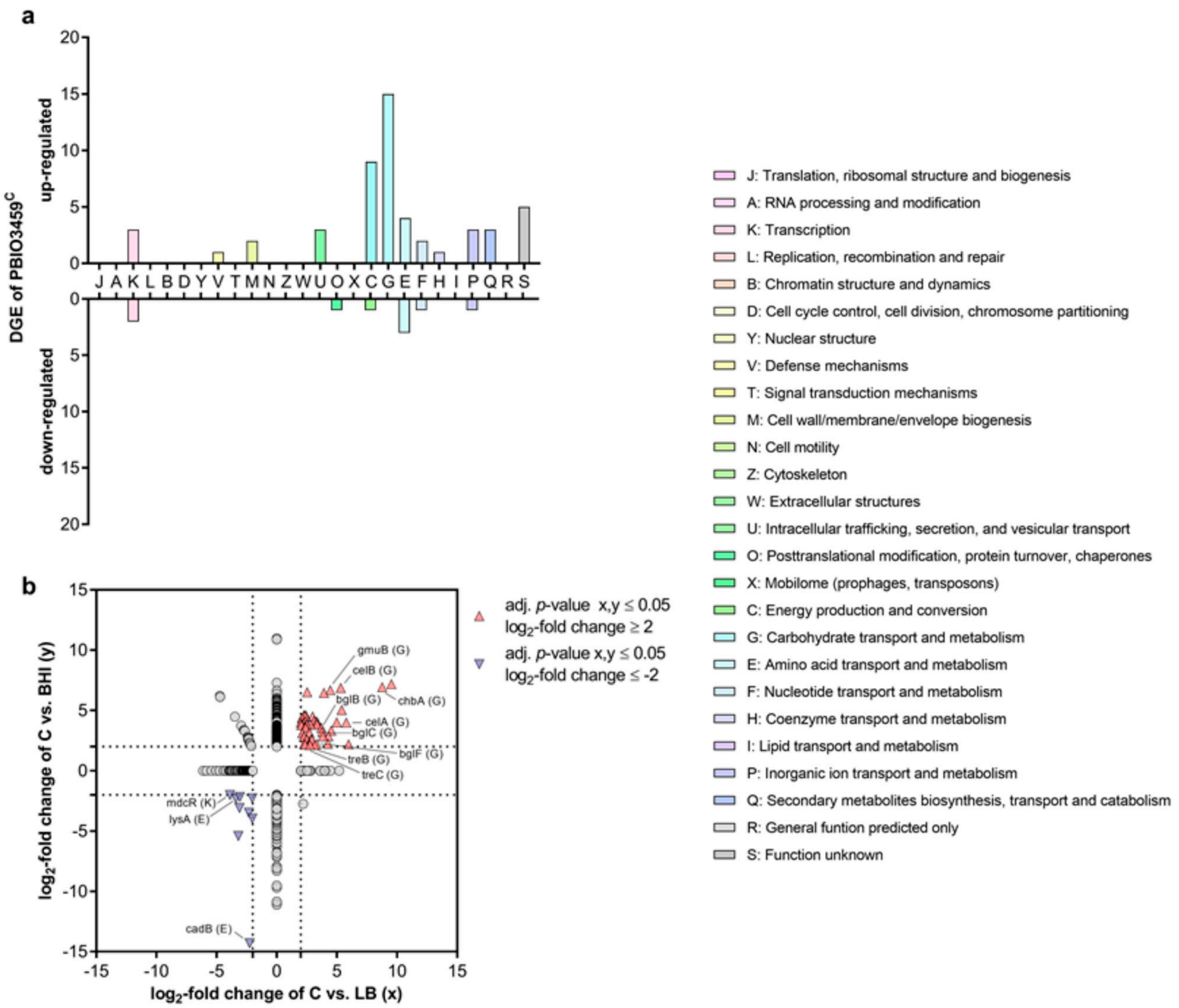

Figure S1. Summary of the differential gene expression. (a) Functional classification of differentially expressed genes of PBIO3459 on chromogenic (C) medium compared to both, LB and BHI medium; based on the COG database. (b) Correlation analysis of C vs. LB (x-axis) and C vs. BHI (y-axis). Similar DGE profiles in the upper-right and lower-left corners indicate transcriptomic changes specifically for chromogenic medium. The most important genes are highlighted and classified according to COG.
