# Supplementary material for: Geno- and Phenotypic Characteristics of a Klebsiella pneumoniae ST20 Isolate with Unusual Colony Morphology
Source: Microorganisms. 2022 Oct 19;10(10):2063. doi: 10.3390/microorganisms10102063 (PMC9606995; doi:10.3390/microorganisms10102063)
Supplement: Supplementary file 1 [file microorganisms-10-02063-s001.zip › Table S1.pdf]

Table S1. Metadata for *K. pneumoniae* ST20 genomes used for phylogenetic analysis. *NA*: not applicable (i.e., no metadata provided).

Sample	E*NA* study accession	Host	Isolation source	Location	Collection date
PBIO3459	PRJEB55904	human	blood	Germany	2021

BioSample	GenBank assembly accession	Host	Isolation source	Location	Collection date
SAMD00129645	GCA_004313545.1	human	blood	Japan	2014
SAMD00196002	GCA_015138775.1	human	*NA*	Japan	2018
SAMEA104208303	GCA_022250695.1	*NA*	*NA*	*NA*	*NA*
SAMEA104208310	GCA_022250615.1	*NA*	*NA*	*NA*	*NA*
SAMEA104208313	GCA_022250575.1	*NA*	*NA*	*NA*	*NA*
SAMEA104208315	GCA_022250535.1	*NA*	*NA*	*NA*	*NA*
SAMEA104208437	GCA_022248755.1	*NA*	*NA*	*NA*	*NA*
SAMEA104567656	GCA_902161435.1	human	faecal	United Kingdom	2018
SAMEA104567807	GCA_900774905.1	*NA*	*NA*	*NA*	*NA*
SAMEA104590273	GCA_022255295.1	human	swab	Cambodia	2013
SAMEA2073120	GCA_022244835.1	human	ET tip	Nepal	2012
SAMEA2073121	GCA_022244795.1	human	ET secreation	Nepal	2012
SAMEA2073310	GCA_022244545.1	human	urine	Nepal	2012
SAMEA2273552	GCA_900084805.1	human	blood	United Kingdom	2002
SAMEA2602992	GCA_000823085.1	*NA*	*NA*	*NA*	*NA*
SAMEA3356962	GCA_020149785.1	*NA*	*NA*	*NA*	*NA*
SAMEA3357023	GCA_904863365.1	*NA*	*NA*	*NA*	*NA*
SAMEA3357158	GCA_904863405.1	*NA*	*NA*	*NA*	*NA*
SAMEA3357253	GCA_904866335.1	*NA*	*NA*	*NA*	*NA*
SAMEA3357421	GCA_904866295.1	*NA*	*NA*	*NA*	*NA*
SAMEA3500082	GCA_900510685.1	human	swab	Slovakia	2014
SAMEA3500088	GCA_900510785.1	human	urine	Slovakia	2014
SAMEA3512056	GCA_900512395.1	human	wound secretion	Germany	2014
SAMEA3515082	GCA_900513115.1	human	blood	Italy	2013
SAMEA3515128	GCA_900515275.1	human	blood	Italy	2013
SAMEA3515130	GCA_900514195.1	human	urine	Italy	2013
SAMEA3515150	GCA_900514815.1	human	blood	Italy	2014
SAMEA3531574	GCA_900172565.1	other	sewage	United Kingdom	2014
SAMEA3538565	GCA_900514055.1	human	urine	Italy	2013
SAMEA3538820	GCA_022291755.1	*NA*	*NA*	*NA*	*NA*
SAMEA3649532	GCA_022290895.1	*NA*	*NA*	*NA*	*NA*
SAMEA3649558	GCA_900517475.1	human	blood	Spain	2013
SAMEA3649665	GCA_900500785.1	human	urine	Greece	2014
SAMEA3649680	GCA_900501315.1	human	puncture fluids	Greece	2013
SAMEA3727631	GCA_900506635.1	human	urine	Belgium	2014
SAMEA3729705	GCA_900507805.1	human	lower respiratory tract secretion	Turkey	2013
SAMEA3729744	GCA_900508505.1	human	urine	Turkey	2014
SAMEA3886655	GCA_022247145.1	*NA*	*NA*	*NA*	*NA*
SAMEA3886656	GCA_022247045.1	*NA*	*NA*	*NA*	*NA*

SAMEA4362606	GCA_900173305.1	human	feces	United Kingdom	2015
SAMEA4362608	GCA_900173335.1	human	feces	United Kingdom	2015
SAMEA4362609	GCA_900173365.1	human	feces	United Kingdom	2015
SAMEA4362668	GCA_900173905.1	human	feces	United Kingdom	2015
SAMEA4531897	GCA_022298775.1	*NA*	*NA*	*NA*	*NA*
SAMEA4531985	GCA_022297115.1	*NA*	*NA*	*NA*	*NA*
SAMEA4532230	GCA_022292975.1	*NA*	*NA*	*NA*	*NA*
SAMEA4807837	GCA_900498415.1	human	*NA*	Germany	2014
SAMEA4807844	GCA_900498585.1	human	*NA*	Germany	2014
SAMEA4807846	GCA_900498385.1	human	*NA*	Germany	2014
SAMEA4807847	GCA_900498595.1	human	*NA*	Germany	2014
SAMEA4807849	GCA_900498475.1	human	*NA*	Germany	2014
SAMEA4807853	GCA_900498405.1	human	*NA*	Germany	2014
SAMEA5048777	GCA_022200005.1	*NA*	*NA*	*NA*	*NA*
SAMEA5049787	GCA_022183305.1	*NA*	*NA*	*NA*	*NA*
SAMEA5050265	GCA_021855905.1	*NA*	*NA*	*NA*	*NA*
SAMEA56276668	GCA_022236885.1	human	*NA*	Ireland	2016
SAMEA56282668	GCA_022236625.1	human	*NA*	Ireland	2016
SAMEA56283418	GCA_022236435.1	human	*NA*	Ireland	2016
SAMEA6657263	GCA_021972375.1	human	urine	Denmark	2018
SAMEA6657273	GCA_021972355.1	human	urine	Denmark	2018
SAMEA6658658	GCA_022175505.1	human	clinical	Cambodia	2016
SAMEA7746285	GCA_916163495.1	human	urine	Netherlands	2010
SAMEA7773563	GCA_021979815.1	human	feces	Norway	2015
SAMEA7773566	GCA_021979795.1	human	feces	Norway	2015
SAMEA7773587	GCA_021979515.1	human	feces	Norway	2015
SAMEA7773609	GCA_021979135.1	human	feces	Norway	2015
SAMEA7773665	GCA_021978415.1	human	feces	Norway	2015
SAMEA7773750	GCA_021977475.1	human	feces	Norway	2015
SAMEA7773776	GCA_021977175.1	human	feces	Norway	2015
SAMEA7773800	GCA_021976895.1	human	feces	Norway	2015
SAMEA7773848	GCA_021976355.1	human	feces	Norway	2015
SAMEA7773889	GCA_021975675.1	human	feces	Norway	2015
SAMEA7773911	GCA_021975395.1	human	feces	Norway	2015
SAMEA7773923	GCA_021975255.1	human	feces	Norway	2015
SAMEA7773931	GCA_021975155.1	human	feces	Norway	2015
SAMEA7773986	GCA_021974415.1	human	feces	Norway	2021
SAMEA7774006	GCA_021974155.1	human	feces	Norway	2015
SAMEA8217956	GCA_021820075.1	human	clinical	Germany	2018
SAMEA8581355	GCA_021895695.1	human	*NA*	Pakistan	2016
SAMEA882006	GCA_022260635.1	human	urine	Australia	2002
SAMEA882046	GCA_022260575.1	human	urine	Australia	2002
SAMEA882199	GCA_022259635.1	human	respiratory	Viet Nam	2003
SAMEA88384168	GCA_022235845.1	human	*NA*	Ireland	2017
SAMEA8948220	GCA_021924145.1	human	blood	Norway	2005
SAMEA8948260	GCA_021921165.1	human	blood	Norway	2005
SAMEA8948265	GCA_021921105.1	human	blood	Norway	2005
SAMEA8948266	GCA_021921125.1	human	blood	Norway	2005
SAMEA8948330	GCA_021925305.1	human	blood	Norway	2001

SAMEA8948347	GCA_021925005.1	human	blood	Norway	2001
SAMEA8948367	GCA_021924805.1	human	blood	Norway	2001
SAMEA8948511	GCA_021919465.1	human	blood	Norway	2009
SAMEA8948530	GCA_021919205.1	human	blood	Norway	2009
SAMEA8948641	GCA_021917925.1	human	urine	Norway	2012
SAMEA8948645	GCA_021917825.1	human	urine	Norway	2012
SAMEA8948731	GCA_021916865.1	human	blood	Norway	2015
SAMEA8948745	GCA_021929555.1	human	blood	Norway	2015
SAMEA8948747	GCA_021929515.1	human	blood	Norway	2015
SAMEA8948752	GCA_021929415.1	human	blood	Norway	2015
SAMEA8948778	GCA_021929135.1	human	blood	Norway	2015
SAMEA8948798	GCA_021928935.1	human	blood	Norway	2015
SAMEA8948817	GCA_021928715.1	human	blood	Norway	2015
SAMEA8948824	GCA_021926005.1	human	blood	Norway	2015
SAMEA8948838	GCA_021928375.1	human	blood	Norway	2015
SAMEA8948853	GCA_021928155.1	human	blood	Norway	2015
SAMEA8948855	GCA_021928135.1	human	blood	Norway	2015
SAMEA8948861	GCA_021928055.1	human	blood	Norway	2015
SAMEA8948862	GCA_021925945.1	human	blood	Norway	2015
SAMEA8948906	GCA_021925825.1	human	blood	Norway	2015
SAMEA8948921	GCA_021927205.1	human	blood	Norway	2015
SAMN02927722	GCA_022304375.1	human	*NA*	Canada	2009
SAMN02927817	GCA_022301875.1	human	*NA*	USA	2006
SAMN03002380	GCA_002895645.2	human	clinical	Netherlands	2012
SAMN03737944	GCA_001038365.1	human	urine	USA	2013
SAMN03853256	GCA_022266615.1	other	retail chicken	USA	2012
SAMN04357353	GCA_022314735.1	human	blood	United Kingdom	2009
SAMN04357362	GCA_022314635.1	human	blood	United Kingdom	2010
SAMN04357385	GCA_022313335.1	human	blood	United Kingdom	2010
SAMN04357387	GCA_022313355.1	human	blood	United Kingdom	2010
SAMN05770822	GCA_022266285.1	human	*NA*	Singapore	2014
SAMN05770868	GCA_022266015.1	human	*NA*	Singapore	2015
SAMN05956096	GCA_001902815.4	human	respiratory	USA	2013
SAMN05960875	GCA_002186545.1	human	blood	Nigeria	2009
SAMN06112183	GCA_022273395.1	human	liver abscess	Singapore	*NA*
SAMN06437422	GCA_022079125.1	human	blood	USA	2012
SAMN06437474	GCA_022089065.1	human	respiratory	USA	2012
SAMN06437481	GCA_022089205.1	human	urine	USA	2012
SAMN06437509	GCA_022091135.1	human	abscess	USA	2012
SAMN06438484	GCA_022069565.1	human	drain	USA	2014
SAMN06546423	GCA_022300095.1	human	sputum	USA	2016
SAMN06629650	GCA_002855175.1	human	*NA*	Colombia	2010
SAMN06629698	GCA_002853815.1	human	*NA*	Colombia	2002
SAMN07310304	GCA_019426495.1	*NA*	hand wash basin	Germany	2004
SAMN07525395	GCA_022286655.1	human	drain	USA	2017
SAMN07525412	GCA_022283905.1	human	blood	USA	2017
SAMN07525424	GCA_022284285.1	human	urine	USA	2017
SAMN07525438	GCA_022285115.1	human	organ	USA	2017
SAMN07525440	GCA_022285065.1	human	urine	USA	2017

SAMN08148173	GCA_014902355.1	human	*NA*	USA	*NA*
SAMN08148239	GCA_014902985.1	human	*NA*	USA	*NA*
SAMN08148240	GCA_014902835.1	human	*NA*	USA	*NA*
SAMN08148299	GCA_014902715.1	human	*NA*	USA	*NA*
SAMN08382725	GCA_022322845.1	human	blood	Singapore	2012
SAMN08382734	GCA_022323505.1	human	blood	Singapore	2012
SAMN08382744	GCA_022323405.1	human	blood	Singapore	2013
SAMN08382755	GCA_022323045.1	human	blood	Singapore	2011
SAMN08382758	GCA_022323105.1	human	blood	Singapore	2011
SAMN09266884	GCA_003195615.1	human	blood	USA	2017
SAMN09266899	GCA_003196655.1	human	blood	USA	2017
SAMN09475209	GCA_019679205.1	other	*NA*	Germany	2017
SAMN09981252	GCA_008121015.1	human	stool	USA	*NA*
SAMN09981274	GCA_008082375.1	human	stool	USA	*NA*
SAMN10058847	GCA_022270315.1	human	blood	Singapore	2015
SAMN10086811	GCA_014840295.1	human	wound	Pakistan	2016
SAMN10241289	GCA_003861085.1	human	patient fluids	USA	2017
SAMN10522466	GCA_004137665.1	*NA*	feces	China	2015
SAMN11579532	GCA_018108585.1	human	wound swab	Malaysia	2016
SAMN11979504	GCA_022174085.1	human	rectal	Singapore	2014
SAMN11979506	GCA_022173985.1	human	rectal swab	Singapore	2013
SAMN11979507	GCA_022173945.1	human	sputum	Singapore	2013
SAMN11979509	GCA_022173405.1	human	rectal swab	Singapore	2014
SAMN11979511	GCA_022173205.1	human	stool/rectal swab	Singapore	2014
SAMN11979515	GCA_022173085.1	human	stool/rectal swab	Singapore	2014
SAMN12109314	GCA_016617225.1	*NA*	blood	*NA*	*NA*
SAMN12109315	GCA_016617195.1	*NA*	blood	*NA*	*NA*
SAMN12109321	GCA_016617095.1	*NA*	blood	*NA*	*NA*
SAMN12109322	GCA_016617015.1	*NA*	blood	*NA*	*NA*
SAMN12109325	GCA_016616945.1	*NA*	blood	*NA*	*NA*
SAMN12109330	GCA_016616935.1	*NA*	blood	*NA*	*NA*
SAMN12109374	GCA_016615935.1	*NA*	blood	*NA*	*NA*
SAMN12109375	GCA_016615895.1	*NA*	blood	*NA*	*NA*
SAMN12109462	GCA_016614335.1	*NA*	blood	*NA*	*NA*
SAMN12109464	GCA_016614245.1	*NA*	blood	*NA*	*NA*
SAMN12212120	GCA_020115175.1	human	blood	Switzerland	2017
SAMN12212290	GCA_020116535.1	human	urine	Switzerland	2017
SAMN12250575	GCA_010598785.1	human	lung	USA	2015
SAMN12250797	GCA_010590075.1	human	lung	USA	2016
SAMN12250803	GCA_010597245.1	human	lung	USA	2016
SAMN12289391	GCA_022132845.1	human	*NA*	Australia	2009
SAMN13153767	GCA_009661155.1	human	blood	Russia	2018
SAMN13301642	GCA_016635585.1	human	blood	Australia	2015
SAMN13829629	GCA_022054415.1	human	rectal swab	Qatar	2018
SAMN13915673	GCA_016643405.1	human	*NA*	Portugal	*NA*
SAMN14008692	GCA_018137325.1	human	*NA*	Mexico	2000
SAMN14008695	GCA_018137275.1	human	*NA*	Mexico	2000
SAMN14379877	GCA_014654945.1	human	*NA*	China	2019
SAMN14609909	GCA_012968365.1	human	blood	South Korea	2016

SAMN14851502	GCA_021842725.1	human	swab	Singapore	2013
SAMN14967546	GCA_014837535.1	human	clinical	USA	2019
SAMN14967661	GCA_014833295.1	human	*NA*	USA	2017
SAMN15148642	GCA_013736155.1	other	wastewater influent	United Kingdom	2017
SAMN15148738	GCA_014103065.1	other	wastewater influent	United Kingdom	2017
SAMN15374758	GCA_017922235.1	human	*NA*	Malawi	2013
SAMN15831847	GCA_014289915.1	human	*NA*	China	2008
SAMN15832080	GCA_014283815.1	human	*NA*	China	2016
SAMN15868508	GCA_022164965.1	human	urine	USA	2016
SAMN16125015	GCA_015356355.1	other	sewage	*NA*	2019
SAMN16357453	GCA_016903255.1	human	*NA*	USA	*NA*
SAMN16427127	GCA_014982705.1	human	stool	Spain	2019
SAMN16614903	GCA_021823485.1	human	bone	USA	2019
SAMN16823204	GCA_022041575.1	human	*NA*	USA	2017
SAMN16823209	GCA_022041735.2	human	rectal	USA	2017
SAMN16824574	GCA_022028395.1	human	*NA*	Singapore	2015
SAMN16824660	GCA_022027115.1	human	*NA*	Singapore	2016
SAMN16842674	GCA_022038895.1	human	trachael aspirate	USA	2019
SAMN16980962	GCA_022033855.2	human	*NA*	USA	2017
SAMN17371801	GCA_018439785.1	human	stool/rectal swab	Germany	2016
SAMN17766485	GCA_022789545.1	other	marine environment	Norway	2019
SAMN17766489	GCA_022789425.1	other	marine environment	Norway	2019
SAMN17766496	GCA_022789315.1	other	marine environment	Norway	2019
SAMN17766503	GCA_022789245.1	other	marine environment	Norway	2020
SAMN17766513	GCA_022789045.1	other	marine environment	Norway	2020
SAMN17766514	GCA_022789005.1	other	marine environment	Norway	2020
SAMN18822993	GCA_021973675.1	human	rectal swab	USA	2021
SAMN18874704	GCA_019930085.1	human	wound	Afghanistan	2013
SAMN18978597	GCA_022551475.1	human	rectal	USA	2017
SAMN19016749	GCA_021953385.1	human	respiratory	USA	2017
SAMN19374585	GCA_018789905.1	other	water	South Africa	2018
SAMN19658408	GCA_021936275.1	human	blood	Canada	2008
SAMN19797021	GCA_019053895.1	human	sputum	China	2016
SAMN19797042	GCA_019053355.1	human	blood	China	2018
SAMN19797043	GCA_019053395.1	human	urine	China	2018
SAMN19855090	GCA_022991025.1	human	blood	USA	2018
SAMN19855117	GCA_022999365.1	human	blood	USA	2018
SAMN19855118	GCA_022984585.1	human	blood	USA	2018
SAMN19855546	GCA_022998685.1	human	stool	USA	2018
SAMN20033658	GCA_021933475.1	human	swab	Australia	2020
SAMN20104173	GCA_021935855.2	human	toe	USA	2021
SAMN20110663	GCA_022012095.1	human	drainage	Germany	2016
SAMN20524809	GCA_023467275.1	human	*NA*	Singapore	*NA*
SAMN20966795	GCA_023037515.1	*NA*	*NA*	Singapore	*NA*
SAMN20967060	GCA_023039755.1	*NA*	*NA*	Singapore	*NA*
SAMN20967074	GCA_023465775.1	*NA*	*NA*	Singapore	*NA*
SAMN20967113	GCA_023036735.1	*NA*	*NA*	Singapore	*NA*
SAMN20967405	GCA_023041155.1	*NA*	*NA*	Singapore	*NA*
SAMN21922876	GCA_021502945.1	human	rectal swab	Pakistan	2016

SAMN22045166	GCA_021904335.1	human	*NA*	USA	2021
SAMN23040723	GCA_023025805.1	human	blood	China	2018
SAMN23224736	GCA_022861205.1	human	*NA*	China	2018
SAMN23521389	GCA_021897075.1	human	*NA*	USA	2021
SAMN24009778	GCA_021262305.1	human	blood	USA	*NA*
SAMN24009794	GCA_021262085.1	human	blood	USA	*NA*
SAMN24020413	GCA_022116155.1	human	*NA*	United Kingdom	2016
SAMN24020516	GCA_022106455.1	human	*NA*	United Kingdom	2016
SAMN24020995	GCA_022146665.1	human	*NA*	United Kingdom	2016
SAMN26101548	GCA_022431305.1	other	milk	USA	2020
SAMN26366690	GCA_022499505.1	human	urine	Australia	2001
SAMN26366696	GCA_022498265.1	human	blood	Australia	2001
SAMN26366712	GCA_022499325.1	human	urine	Australia	2001
SAMN26366729	GCA_022500005.1	human	urine	Australia	2002
SAMN26368956	GCA_023033685.1	human	urine	Australia	2020
SAMN26369082	GCA_023028205.1	human	sputum	Australia	2020
SAMN26369217	GCA_023030245.1	human	urine	Australia	2020
SAMN26369305	GCA_023033635.1	human	blood	Australia	2020
SAMN26417635	GCA_022470475.1	human	sputum	USA	2021
SAMN26976180	GCA_023206495.1	human	rectal swab	USA	2017
SAMN26976209	GCA_023311735.1	human	rectal swab	USA	2017
SAMN26976276	GCA_023311615.1	human	rectal swab	USA	2017
SAMN26976323	GCA_023207175.1	human	rectal swab	USA	2018
SAMN26976365	GCA_023207895.1	human	rectal swab	USA	2018
SAMN26976393	GCA_023462755.1	human	rectal swab	USA	2018
SAMN26976425	GCA_023314275.1	human	rectal swab	USA	2018
SAMN28097314	GCA_023829615.1	human	sputum	Thailand	2017
SAMN28743183	GCA_023584565.1	human	rectal swab	Australia	2019
